# Variants in *NR6A1* cause a novel oculo-vertebral-renal (OVR) syndrome

**DOI:** 10.21203/rs.3.rs-5375105/v1

**Published:** 2024-11-15

**Authors:** Uma M. Neelathi, Ehsan Ullah, Aman George, Mara I. Maftei, Elangovan Boobalan, Daniel Sanchez-Mendoza, Chloe Adams, David McGaughey, Yuri V. Sergeev, Ranya AI Rawi, Amelia Naik, Chelsea Bender, Irene H. Maumenee, Michel Michaelides, Tun Giap Tan, Siying Lin, Rafael Villasmil, Delphine Blain, Robert B. Hufnagel, Gavin Arno, Rodrigo M. Young, Bin Guan, Brian P. Brooks

**Affiliations:** 1. Ophthalmic Genetics & Visual Function Branch, National Eye Institute, National Institutes of Health, Bethesda, MD 20892.; 2. UCL Institute of Ophthalmology, University College, London, London, UK; 3. Harkness Eye Institute, Columbia University, 622 W 168^th^ St., New York, NY 10032; 4. Moorfields Eye Hospital, NHS Foundation Trust, London, UK; 5. Torbay Hospital, Torbay and South Devon NHS Foundation Trust, Devon, UK; 6. Flow Cytometry Core, National Eye Institute, Bethesda, MD 20892; 7. Center for Integrated Health Care Research, Kaiser Permanente Hawai’i; Hawai’i Permanente Medical Group, Honolulu, HI; 8. Greenwood Genetic Center, Greenwood, SC 29646; 9. Center for Integrative Biology, Universidad Mayor, Santiago, Chile; 10. To whom correspondence should be addressed

## Abstract

Colobomatous microphthalmia is a potentially blinding congenital ocular malformation that can present either in isolation or together with other syndromic features. Despite a strong genetic component to disease, many cases lack a molecular diagnosis. We describe a novel autosomal dominant oculo-vertebral-renal (OVR) syndrome in six independent families characterized by colobomatous microphthalmia, missing vertebrae and congenital kidney abnormalities. Genome sequencing identified six rare variants in the orphan nuclear receptor gene *NR6A1* in these families. We performed in silico, cellular and zebrafish experiments to demonstrate the *NR6A1* variants were pathogenic or likely pathogenic for OVR syndrome. Knockdown of either or both zebrafish paralogs of *NR6A1* results in abnormal eye and somite development, which was rescued by wild-type but not variant *NR6A1* mRNA. Illustrating the power of genomic ascertainment in medicine, our study establishes *NR6A1* as a critical factor in eye and vertebral development and a pleiotropic gene responsible for OVR syndrome.

## Introduction

Uveal coloboma is a congenital ocular malformation caused by failure of the ventral optic fissure to close during early eye morphogenesis and is usually considered on a phenotypic continuum with microphthalmia and anophthalmia ^1–5^. A rare condition^6–11^, coloboma may nonetheless account for up to 10% of childhood blindness^12^. Although significant progress has been made in identifying genes associated with syndromic and non-syndromic coloboma, the yield of diagnostic testing remains low, especially for isolated, non-syndromic coloboma, suggesting other genes are yet to be discovered^13–15^. To identify novel coloboma genes, the National Eye Institute has conducted a natural history study since 2006, on the genetics of coloboma that includes systematic deep phenotyping of probands and first-degree family members. We have previously identified a novel syndrome characterized by missing vertebrae (in the thoracic and/or lumbar spine) and uveal coloboma, inherited in an autosomal dominant fashion with incomplete penetrance and variable expressivity^16^.

We identified structural and sequence variants in the transcription factor gene *NR6A1* (*Nuclear receptor subfamily 6, group A, member 1,* OMIM*602778) in three families by genome sequencing (GS). These results were extended via analysis of the Genomics England 100,000 Genomes Project (UK100KGP), where three additional individuals with microphthalmia/anophthalmia/coloboma were identified ^17^.

Originally termed germ cell nuclear factor (*GCNF*)/retinoid receptor-related testis-associated receptor (*RTR*), *NR6A1* is an orphan member of the nuclear hormone receptor family of transcription factors, often acting as a transcriptional repressor. *NR6A1* is highly expressed in embryonic and other stem cells from various tissues (especially testes) and is repressed upon differentiation^18^. *NR6A1* plays an important role in somite and subsequent vertebral development in mice, and in livestock species it is correlated with vertebral number ^19–22^. To our knowledge there are no reports on the role of *NR6A1* in eye or kidney development.

Here we described a novel autosomal dominant oculo-vertebral-renal (OVR) syndrome caused by variants in the orphan nuclear receptor gene *NR6A1*, supporting the pathogenicity of variants through a combination of in silico, in vitro and in vivo investigations. To our knowledge, this is first mendelian trait in humans characterized by missing vertebrae.

## Methods

### Patients and clinical studies

Complete eye examinations and genetic testing at the National Eye Institute (NEI) were conducted under IRB-approved clinical protocols (NCT01778543, NCT01087320, NCT02077894, www.clinicaltrials.gov). Probands underwent systemic testing as clinically indicated, which include physical exam, kidney ultrasound, routine blood chemistries, audiology, and spine x-ray. Eye examinations included age-appropriate testing of visual acuity, refraction, ocular motility/alignment, slit lamp exam, dilated fundus exam and ophthalmic photography. Specific informed consent for exome/genome sequencing was obtained under an IRB-approved protocol along with pre- and post-test genetic counseling (NCT02077894). Family COL005 and COL034 were previously reported as Family 1 and 2, respectively, without molecular characterization and detailed individual phenotyping data^16^. For patients and relatives recruited from the Genomics England 100,000 Genomes Project (UK100KGP), informed consent for whole genome sequencing (GS) was obtained in accordance with approval from the HRA committee East of England-Cambridge south (REC 14/EE/1112)^17^. Details of gene/protein expression, bioinformatic, molecular modeling, and zebrafish experiments are described in detail in Supplemental Materials and Methods.

## Results

### Variants in NR6A1 cause an oculo-vertebral-renal (OVR) syndrome

**Fig. 1:**
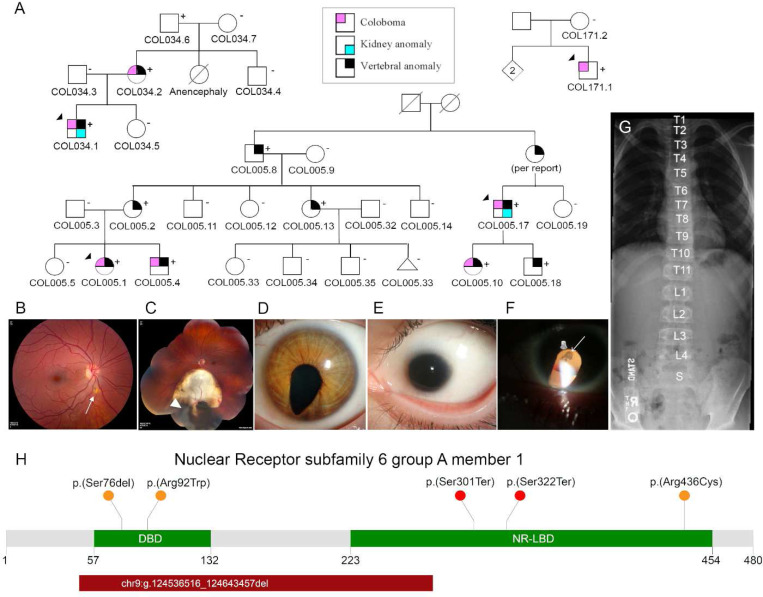
Phenotypes associated with variants in *NR6A1*. **A**. Pedigrees of three families (COL005; COL034; COL171) from the NEI cohort demonstrating coloboma with or without microphthalmia and cataract, missing vertebrae, and congenital renal anomalies. Inheritance is autosomal dominant with incomplete penetrance and variable expressivity. **B**. Linear pigmentary disturbance representing a *forme fruste* of coloboma (arrow) in COL005.1 (right eye). **C**. Larger chorioretinal coloboma in the left eye of COL005.1 demonstrating a retinal tear in the far periphery (arrowhead). **D**. Iris coloboma of the left eye of COL005.10. **E.** Microphthalmia of the left eye in COL034.1. **F**. Retroillumination image of the left eye of COL171.1 demonstrating iris coloboma and posterior subcapsular cataract (open arrow). **G**. Spine x-ray of COL005.4 demonstrating 11 thoracic (normal 12) and 4 lumbar (normal 5) vertebrae. **H**. Schematic of NR6A1 variants detected in the NEI and UK Genomics England cohorts. +, individual with variant; -, individual without variant. DNA binding domain (DBD) and putative nuclear receptor ligand binding domain (NR-LBD) are noted.

We identified three rare *NR6A1* variants in three families affected by uveal coloboma (COL005, COL034, COL171) with or without microphthalmia, cataract, and missing vertebrae through genome sequencing. In cases where multiple generations are affected, transmission is autosomal dominant with incomplete penetrance and variable expressivity (Fig. 1A). Clinical data for all the participants with a positive molecular result in shown Table 1. No other candidate pathogenic variants in *NR6A1* were identified in the NEI coloboma cohort consisting of a total of 224 probands (66 analyzed by genome sequencing, 57 by exome sequencing, and 101 by amplicon sequencing).

The proband of the family (COL005.1) presented at age 14 with bilateral uveal colobomas (Fig. 1B, C). Family history was notable for a younger brother (COL005.4), a second cousin (COL005.10) and first cousin once removed (COL005.17) with uveal coloboma. The deletion breakpoints were in intron 2 and 6 removing the coding sequence for amino acids (aa) Ile48-Gly275 and likely causing a frameshift (p.Ile48Asnfs*3, Fig. 1H). The status of the heterozygous deletion was determined by breakpoint PCR among family members available, which revealed complete segregation with the missing vertebrae with an estimated LOD score of 3.6 (Fig. 1A, Supplementary Fig. S1). Four family members were also affected by coloboma in addition to missing vertebra, of which one (COL005.17) also had, by report, only one kidney. The proband of family (COL034.1) presented at age 11 months with bilateral uveal colobomas and microphthalmia OS (Fig. 1E). Genome sequencing revealed a heterozygous c. 274C>T p. (Arg92Trp) variant in *NR6A1*, which was found in the affected mother and essentially unaffected grandfather (Fig. 1A, H). The proband of family (COL171.1) presented at age 36 with bilateral colobomatous microphthalmia affecting the iris, retina/choroid and optic nerve. Slit lamp exam was notable for bilateral microcornea, bilateral posterior subcapsular and nuclear cataracts and missing zonules inferiorly OU (Fig. 1F). Genome sequencing revealed a heterozygous c.1306C>T p.(Arg436Cys) variant in the proband which was absent in his unaffected mother (Fig. 1H). Detailed study of the probands and their family members available for evaluation, were described in clinical vignettes in the extended data. No convincing pathogenic variants in known coloboma genes were identified in any of these subjects.

### Genome-first approach for NR6A1 variants corroborates MAC phenotypes

We performed an unbiased disease association analysis of rare pLoF variants using the UK100KGP dataset^17^. After removing variants resulting from calling artifacts or mis-annotation, only three pLoF variants were found in the cohort with approximately 126,700 alleles (Supplementary Table 1, Supplementary Fig. 2). We found three probands, Proband (A1) presented at age 30 with bilateral chorioretinal coloboma (*forme fruste* OD) and OS coloboma of the optic nerve (Supplementary Fig. 2B). Genome sequencing revealed a heterozygous c.965_980del p.(Ser322Ter), present in both the proband and her unaffected father. Proband (B1) presented at the age of 29, with a severe form of bilateral microphthalmia with a vestigial remnant of eye, delayed motor development, intellectual disability, abnormal behavior, and schwannoma. This proband carried a heterozygous c.902G>A p.(Trp301Ter) variant. These two nonsense variants are expected to cause loss of protein function either through nonsense-mediated decay or truncation of the putative nuclear receptor ligand binding domain (NR-LBD, Fig. 1H). Proband D (Supplementary Table 1) had a disorder of sex development carried variant c.288dup p.(Cys96TrpfsTer4), which was absent in either parent. His father was also affected with a disorder of sex development, suggesting that the *NR6A1* variant is likely not associated with the condition.

The UK100KGP MAC cohort, which consists of 215 probands, was queried for rare missense and in-frame insertion/deletion variants. Proband C1, presented at the age of 25 with bilateral microcornea and coloboma affecting the iris, choroid/retina, and optic nerve. One brother had a similar condition by report. Both parents, and the two other siblings of the proband had no history of coloboma by report. Genome sequencing revealed a heterozygous variant c.227_229del p.(Ser76del) present in the proband (Fig. 1H, Supplementary Table 1). This variant leads to an in-frame deletion of a serine within the Zn-finger motif. Within the three MAC patients we report, no candidate pathogenic variants were found in the known MAC genes present in the current Genomics England PanelApp (ocular coloboma v1.47, anophthalmia or microphthalmia v1.51, structural eye disease v3.79). Thus, these cases further support that rare variants in *NR6A1* can cause MAC with reduced penetrance.

### Molecular Modeling Suggests Missense Variants Disrupt Important Intramolecular Interactions

The NR6A1 amino acid sequence is well-conserved between human, mouse, and zebrafish; specifically, the residues Ser76, Arg92 and Arg436 are conserved across multiple species (Supplementary Fig. 3). To understand the effects, missense variants had on protein stability and function, we created an *in silico* model of a complex of NR6A1 with DNA (Supplementary Fig. 4A). The AlphaFold model of NR6A1 is shown by the composition of Zn-finger (residues 60–172) and NR_LBD (residues 246–480) domains shown in orange and green, respectively. The rest of the model shown in gray is predicted as an irregular structure by AlphaFold. The locations of variants R92W and R436C are labeled. In wild-type (WT) NR6A1, a positively charged arginine residue 92 interacts with negatively charged DNA (Supplementary Fig. 4B). The R92W variant replaces the R92 residue with hydrophobic tryptophan (W), interrupting the electrostatic interaction with DNA and possibly reducing the DNA binding of the Zn-finger motif. The R436C variant affects the putative nuclear receptor ligand binding domain NR_LBD. In NR6A1, hydrogen atom 1HH2 of arginine R436 closely interacts with the oxygen atom of glutamic acid E388 (Supplementary Fig. 4C). The Variant R436C breaks this bond creating a cysteine residue instead of an arginine. In this variant domain, residues C443, C391, and C422 are distanced at 8–12 Å from C436. In the native state of protein, the reduced state of cysteine residues is protected. However, any oxidative damage could cause the formation of non-native disulfide bonds affecting protein structure and ligand binding.

### Missense variants alter NR6A1 protein subcellular localization

**Fig. 2:**
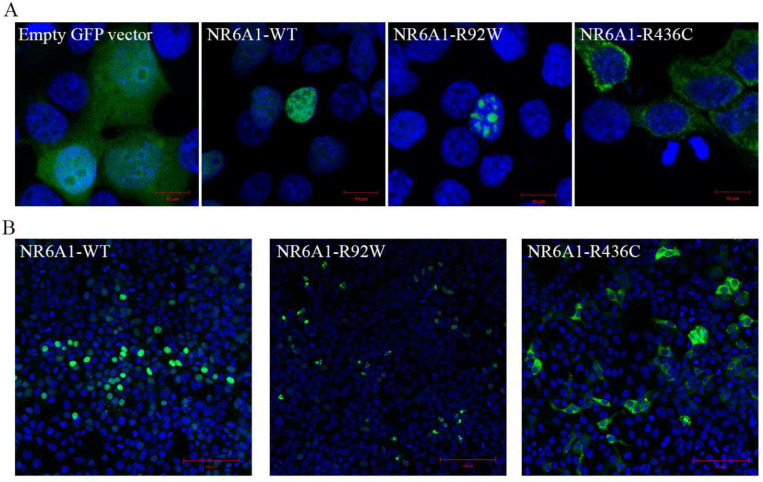
Subcellular localization of wild-type (WT) and mutant forms of NR6A1. NR6A1 variant localization pattern was studied by overexpression in HEK293 cells and representative high magnification (63X) images are shown from three different trials (A). Scale bar = 10 μm. The localization pattern for the WT and the two variant isoforms was observed to be consistent across three transfection experiments. (Cells counted: WT=387, R92W=350 and R436C=217). Scale bar = 100 μm.

To study the functional impact of the missense variants on protein localization in the cell, the R92W and R436C mutations were introduced in WT *NR6A1* cDNA fused to a GFP coding sequence. All experiments were performed in context to the *NR6A1* isoform NM_033334.4 and repeated at least three times. Transfection efficiencies were between 50–60% and not significantly different between WT and variant constructs as analyzed by flow-cytometry and Western blotting (Supplementary Fig. 5, 6). The WT-*NR6A1* when over-expressed in HEK293 cells was consistently observed to localize in the nucleus (Fig. 2A, B), consistent with a previous report. The R92W variant, although nuclear, was not uniform in its distribution, forming apparent protein aggregates. In contrast, the R436C variant localized exclusively in the cytoplasm (Fig. 2A). The above-described localization pattern of the WT and variant isoforms was consistent in all transfected cells and across multiple rounds of transfection. Taken together these results suggest that both missense variants likely interfere with NR6A1 function due to improper subcellular localization.

### Expression pattern of mouse and zebrafish NR6A1 homologs suggests a role in early eye, kidney, and somite development

**Fig. 3:**
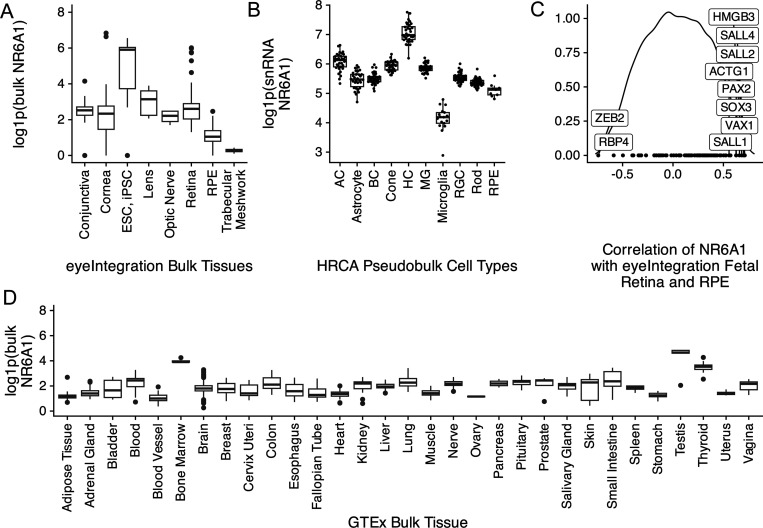
A. Comparative levels of NR6A1 from publicly available bulk human tissue RNA-sequencing (RNA-Seq) datasets accessed on the eyeIntegration website (https://eyeintegration.nei.nih.gov/). On average, expression is higher in embryonic and induced pluripotent stem cells (ESC, iPSC, respectively) than in adult ocular tissues. B. In adult retina, expression of *NR6A1* is highest in horizontal cells (HC) compared to other cell types in the Human Retinal Cell Atlas (HRCA) (AC, amacrine cell; BC, bipolar cell; MG, Müller glia; RGC, retinal ganglion cell; RPE, retinal pigment epithelium). C. Correlation of NR6A1 expression with fetal retina and RPE RNA-Seq data demonstrates association with several known coloboma associated genes (boxed labels). D. Among systemic tissues, *NR6A1* is expressed most highly in bone marrow and testes.

Analysis of bulk RNA-Seq datasets from ocular and non-ocular tissues demonstrates modest expression of *NR6A1* in most tissues and relatively higher levels of expression in embryonic stem cells/induced pluripotent stem cells (compared to adult ocular tissues) and in bone marrow and testis systemically (Fig. 3A, D)^23,24^. In the Human Retinal Cell Atlas single nucleus RNA-Seq dataset *, NR6A1* is highly expressed in adult horizontal cells and low in microglia and RPE (Fig. 3B)^25^. Expression of *NR6A1* is strongly correlated (>5 fold enrichment, p = 0.0024) with that of other coloboma-associated genes in fetal ocular tissues (Fig. 3C). This strength of enrichment was not seen in Genotype-Tissue Expression (GTEx) body tissue (p=0.361) or adult eye tissue (p=0.451) ^23,24^. We note that several of the enriched genes-*SALL4* (Duane-Radial Ray Syndrome), *PAX2* (Papillorenal syndrome), *ACGT1* (Baraitser-Winter Syndrome 2), *SALL1* (Townes-Brocks Syndrome 1) can also present with congenital renal anomalies.

To establish plausible causation for *NR6A1* variants, we studied the embryonic expression of its orthologs in mouse and zebrafish model systems at developmentally relevant time points. Previous work has demonstrated widespread expression of *Nr6a1* in mouse at E8.5 and E9.5 (including the optic vesicle) that becomes nearly undetectable by E12.5. To study expression in the optic cup around the time of optic fissure closure, we used a probe that detects all validated transcripts of mouse *Nr6a1* at embryonic day 10.5 (E10.5, early optic cup) and E11.5 (time of optic fissure closure). At E10.5, we noted diffuse low-level expression throughout the early optic cup and surrounding tissues that becomes significantly decreased by the time optic fissure closure commences (E11.5) (Supplementary Fig. 7).

**Fig. 4:**
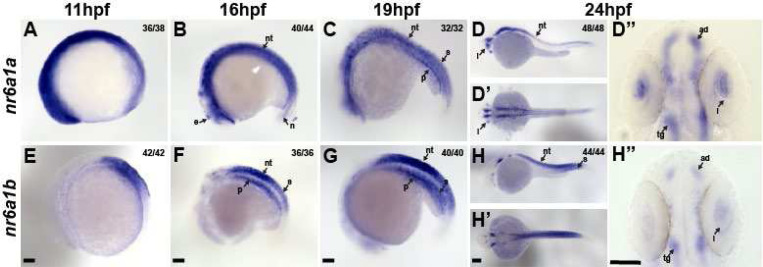
Expression pattern of *nr6a1a* and *nr6a1b* paralogs in zebrafish. *nr6a1a* is expressed ubiquitously at 11 hours post-fertilization (hpf) (A). By 16–19 hpf (B, C) expression is present in the somites (S), neural tube (NT), and notochord (N). At 24 hpf, expression remains in the NT but is decreased in the S and N. Expression in the lens (L) is first noted at 19 hpf and is particularly prominent by 24 hpf (D-D’’). *nr6a1b* expression at 11 hpf is anterior trunk, localizing to neural tube and somites from 16 hpf (F) and 19 hpf (G). At 24hpf (H-H’’) it remains expressed in the neural tube and somites, with faint expression can be seen in the lens. All embryos are oriented in a lateral view, anterior to the left and dorsal up, except D’ and H’ shown in dorsal views. Scale bar = 100 μM. e-epiphysis, l-lens, p-pronephros, n-notochord, s-somite, nt-neural tube, ad-anterior diencephalon, tg-tegementum.

In zebrafish, *nr6a1* has two paralogs, *nr6a1a* and *nr6a1b,* both of which are maternally expressed. At 11hpf, when the optic vesicle evaginates, *nr6a1a* is widely expressed throughout the embryo, especially rostrally, showing less expression towards the posterior embryo axis (Fig. 4A). At 16hpf, *nr6a1a* remains widely expressed becoming restricted to the ventral regions of the brain, epiphysis, periocular tissues, heart and in the notochord and neural tube (Fig. 4B). Notably, *nr6a1a* expression is absent from the neural-mesodermal progenitor region in the tail of zebrafish embryos, consistent with its role in the trunk differentiation program. By 19hpf the expression appears to decrease overall but remains present in the ventral brain regions, notochord, somites, and the pronephric duct (Fig. 4C). At 24hpf, expression is prominent in the anterior diencephalon, tegmentum, midbrain, and along most of the length of the embryo in the neural tube; interestingly, expression is nearly absent from the neural retina and retina pigmented epithelia but is prominent in the lens (Fig. 4D–D’’), a pattern not noted in the mouse *Nr6a1* expression. After 26hpf and up to 72hpf we observed no detectable *nr6a1a* expression, consistent with published single-cell mRNA expression during zebrafish development.

Unlike *nr6a1a*, *nr6a1b* expression at 11hpf is limited to a patch in the posterior neuroectoderm of the embryo but excluded from the most caudal region (Fig. 4E). At 16hpf and 19hpf, *nr6a1b* expression is prominent in the neural tube, somites, and pronephric duct and, like *nr6a1a,* is excluded from the neural-mesodermal progenitor region in the tail (Fig. 4F, G). By 24hpf, expression is decreased in most tissues but remains in the tegmentum, cranial ganglia, neural tube, and somites in the distal region of the trunk (Fig. 4H–H’’). By 36hpf and through 72hpf, *nr6a1b* is notably expressed in the developing lens, brain, and cranial ganglions. (Supplementary Fig. 8).

### Morpholino knockdown of zebrafish nr6a1a/nr6a1b recapitulates human phenotypes which are not rescued by pathogenic variant mRNA

**Fig. 5:**
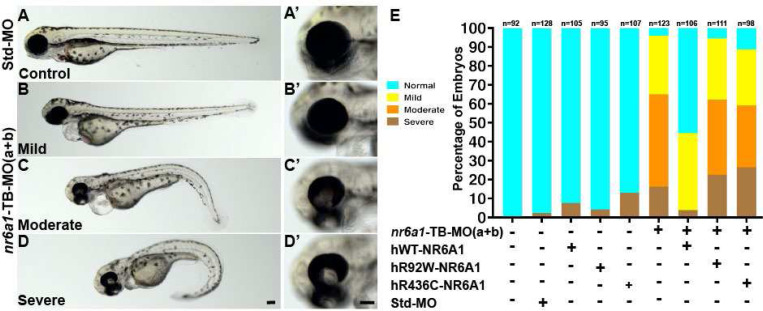
Rescue of *nr6a1+nr6a1b* zebrafish morphant phenotypes with wildtype and mutant human NR6A1 mRNA: Controls (A, A’) have a straight body axis and the optic fissure (OF) is closed. The ***nr6a1+nr6a1b*** morphants that have a mild phenotype (B, B’) have close to a normal body with microphthalmia and heart edema; a moderate phenotype (C, C’) with a slightly bent body axis with smaller eyes, coloboma and a severe heart edema; and severe morphants (D, D”) have a curved body axis with smaller eyes, coloboma and heart edema. The morphant phenotype was rescued when the morpholinos were co-injected along with the human-*NR6A1*-wild type mRNA. However, there was no significant rescue in the morphant phenotype when the morpholinos were injected with either R92W or R436C human disease-causing variants (E). Morpholinos were injected at 0.75 ng each (1.5 ng total). Scale bar = 100μM

All the morpholinos experiments are carried out following the guidelines set forth for their use in zebrafish. To test the functional consequences of *nr6a1a* and *nr6a1b* knockdown, we designed translation (TB) and splice blocking (SB) morpholinos for each paralog of the gene. Morphants were divided into four phenotypes: normal, mild (normal/near normal body axis w/ microphthalmia), moderate (slightly shortened and mildly curved body axis, microphthalmia ± coloboma and heart edema) or severe (significantly shortened and curved body axis, microphthalmia ± coloboma, heart edema) (Fig. 5A–D, Supplementary Fig. 9, 10). Embryos were scored at 72 hpf (after optic fissure closure and initial stages of eye growth are normally completed) to ensure microphthalmia/coloboma represents a true phenotype and not because of developmental delay or undergoing growth compensation.

Knockdown of *nr6a1a* (Supplementary Fig. 9E) or *nr6a1b* (Supplementary Fig. 10E) with either TB-MO or SB-MO resulted in a significant number of moderate/severe phenotypes with few mild phenotypes. Although the effect of TB-MO and SB-MO were similarly potent for *nr6a1b* knockdown, the SB-MO had a stronger effect than the TB-MO for *nr6a1a*. SB-MO knockdown of the gene was validated for both paralogs by reverse transcription-PCR experiments (Supplementary Fig. 9F, 10F). The phenotypic spectrum was not affected by co-injection with p53 morpholino, suggesting widespread cell death was not the primary cause of our observations (data not shown).

Overexpression of 100 pg of human *NR6A1* mRNA in zebrafish shows no overt phenotype (Supplementary Fig. 11A, B). Co-injection of 2 ng and 1.25ng of nr6a1a and nr6a1b, TB-MO respectively along with 100 pg of WT human mRNA (hWT-*NR6A1*), resulted in a rescue, with over 60% embryos exhibiting a normal/control-injected phenotype (Supplementary Fig. 9G, S10G). In contrast, co-injection with either hR92W or the hR436C missense variants of *NR6A1* identified in coloboma patients were significantly less effective in rescuing the zebrafish *nr6a1a/b* knockdown, indicating that the missense variants are deleterious (Supplementary Fig. 9G, 10G).

To study the effect of knocking down both *nr6a1a* and *nr6a1b* zebrafish paralogues, we co-injected 0.75 ng of TB-MO for each paralog (1.5 ng total), resulting in a similar spectrum of phenotypes compared to the knockdown of individual paralogues (Fig. 5A–D, A’–D’). While injection of TB-MO resulted in >60% embryos having a moderate or severe phenotype, co-injection of 100 pg hWT-*NR6A1* mRNA, resulted in >50% normal embryos. Neither the hR92W or hR436C *NR6A1* mRNAs resulted in significant rescue, confirming the pathogenicity of these variants (Fig. 5E). Injection of 0.75 ng of either *nr6a1a* TB-MO or *nr6a1b* TB-MO resulted in a significantly milder phenotype, suggesting that co-injection of these had at least an additive phenotypic effect in the combined MO injection experiment (Supplementary Fig. 12).

Because a prior study reported that both overexpression and loss-of-function of *nr6a1* can result in developmental phenotypes in *Xenopus laevis*, we also evaluated the effect of injection of human *NR6A1* mRNA on zebrafish development. Overexpression of 150 pg of *hNR6A1* mRNA resulted in microphthalmia and heart edema with a straight body axis (n=91/108) (Supplementary Fig. 11C). At 200 pg, overexpression of *hNR6A1* mRNA, phenotypes were more severe including colobomatous microphthalmia, heart edema and a bent body axis (n=60/92), with 26% (n=24/92) exhibiting noticeable shortening and loss of chevron-shaped somites (Supplementary Fig. 13); a minority of embryos (n=8/92) developed no discernible eyes (Supplementary Fig. 11F). Taken together, these experiments demonstrate that normal zebrafish eye development is sensitive to *nr6a1* dosage and both reduced and increased *nr6a1* expression result in developmental phenotypes analogous to human colobomatous microphthalmia.

## Discussion

Here we describe six *NR6A1* variants that cause an autosomal dominant syndromic form of colobomatous microphthalmia and missing vertebrae with or without congenital kidney abnormalities, that we term OVR syndrome. As with many other cases of syndromic and non-syndromic microphthalmia/coloboma, the OVR syndrome show incomplete penetrance and variable expressivity ^1^. By 2015 ACMG/AMP variant interpretation criteria, we considered chr9:g.124536516_124643457del pathogenic (criteria: PVS1, PP1_Strong, PM2) and other MAC-associated variants likely pathogenic (criteria: Ser76del, PM1, PM2, PM4, PP3; Arg92Trp, PS3, PM1, PM2, PP3; Arg436Cys, PS3, PM2, PP3; Ser301Ter & Ser322Ter, PVS1, PM2). Thus, *NR6A1* variants were causative among 1.3% - 1.4% families in two independent patient cohorts (3 out of 224 in the NEI coloboma cohort and 3 out of 215 in the MAC cohort in the UK100KGP).

The NEI study, which specifically recruits patients with coloboma/microphthalmia, performs extensive phenotypic analysis on probands including complete eye examination, kidney ultrasound, neuropsychological testing, physical exam/dysmorphology exam, spine x-ray, routine bloodwork/urinalysis, ECHO (in the presence of a murmur), and audiology. Additional testing (e.g., brain MRI) may be performed on an as needed basis. In addition, all available first-degree relatives undergo a complete dilated fundus exam. As such, we have greater certainty that a patient is truly unaffected, say, by coloboma, rather than being simply asymptomatic. Indeed, the mother of the proband in family COL034 (COL034.2), for example, was visually asymptomatic and unaware of a *forme fruste* of coloboma or a missing thoracic vertebra prior to her exam with us. Conversely, the Genomics England database spans an entire population in a gene and phenotype agnostic manner but may contain incomplete or unrelated phenotypic information. As such, phenotypes such as intellectual disability (Individual B1, Supplementary Table 1) may be spurious associations or may be uncommon manifestations of an *NR6A1*-related syndrome. Confirmation of these and other possible phenotypes awaits description of additional cases. We include congenital renal disease as part of this new syndrome not only because two individuals in two separate pedigrees exhibited these phenotypes, but also because Rasouly et al. have simultaneously identified presumed loss-of-function variants in thirteen individuals with congenital renal abnormalities, with or without congenital eye abnormalities, providing further validation of our findings (personal communication).

The genotypes and functional data we present suggest haploinsufficiency as the primary mechanism of disease, although we cannot rule out that missense variants may have other, dominant-negative effects, by dimerizing with the wildtype protein or interacting with other transcription regulators. The differences in subcellular localization of the two missense variants in *NR6A1* may indeed hint at more than one mechanism of disease.

The early expression of *NR6A1* homologs in mouse and zebrafish are consistent with the previous data and suggest that the colobomatous microphthalmia observed in our patients may result from effects on early eye morphogenesis rather than a defect in optic fissure closure *per se*. However, given the expression of *nr6a1a/nr6a1b* in the lens vesicle in zebrafish, a non-cell autonomous effect on optic fissure closure cannot be excluded. In fact, evidence from Mexican surface and cave fish (*Astyanax mexicanus)* experiments show that early neural retina development and maintenance relies on a healthy lens ^26,27^.

Recently, *NR6A1* has been shown to be important for somite development and, consequently, vertebral number, thus strengthening the phenotyping link with missing vertebrae we describe in humans^19–22,28^. Vertebrae differentiate from somites which develop their stereotyped segmentation pattern in an anterior to posterior progression during early development, with successive *HOX* genes specifying different regions of the spine via a process called temporal collinearity. Homozygous germline inactivation of *Nr6a1* in mice results in embryonic lethality around E10.5 with cardiovascular, neural tube and hindgut abnormalities as well as fewer somites (13, rather than the normal 25). In S*us domesticus* (pig), *NR6A1* was identified as a quantitative trait locus for vertebral number, which is known to vary between breeds^19,21^. In *Equus assinus* (donkey), an *NR6A1* intronic polymorphism is associated with body size/vertebral number and a single nucleotide polymorphism in exon 8 is associated with the number of lumbar vertebrae in Kazakh sheep ^20,28^. In developing *Xenopus*, *NR6A1* is expressed in late tailbud and neurula stages; overexpression results in posterior defects and disturbed somite formation, while expression of a dominant negative form of the receptor results in abnormal neural tube differentiation, loss of head structure including eyes, and downregulation of a retinoic acid receptor (RARγ2) anteriorly. Retinoic acid treatment of embryos upregulates expression of NR6A1, increasing primary neurogenesis via factors such as NeuroD, XDelta1 and x-ngnrl. Retinoic acid is a known and important regulator of both ocular and kidney development ^29,30^; whether retinoic acid receptor signaling is disrupted in model systems of *Nr6a1/nr6a1* is currently under investigation. However, all phenotypes previously observed when modulating the activity of *NR6A1* in animal models are consistent with the developmental defects in the eyes, kidneys, and vertebrae that we observe in patients carrying deleterious mutations in NR6A1.

In conclusion, genome sequencing identified novel *NR6A1* variants in three unrelated families which are associated with a novel OVR syndrome, these findings were further corroborated in an independent cohort using a genome first approach. Using *in silico* prediction and molecular studies we demonstrated that these highly conserved variants disrupt NR6A1 protein structure leading to mis-localization at the cellular level. We further demonstrated enrichment of coloboma-associated genes with *NR6A1* in fetal, but not adult tissues. Expression of *NR6A1* homologs in mouse and zebrafish embryos suggests disease relevant tissue-specific gene expression pattern. This was further confirmed by *in vivo* experiments where the knockdown of zebrafish *nr6a1a and nr6a1b* resulted in ocular and systemic phenotypes that were partially rescued with WT human *NR6A1* mRNA but not with the two variants tested. This data implicates the human *NR6A1* gene variants with the OVR syndrome.

## Extended Data

### COL005

The proband of family COL005 (COL005.1) presented at age 14 with bilateral uveal colobomas (Figure 1B, C). Past ocular history was remarkable for strabismus surgery and amblyopia OS treated with patching. Past medical history was notable for a “small jaw requiring minor reconstruction for proper dentition”, “abnormal enamel to teeth, requiring capping”, and speech therapy. Family history was notable for a younger brother (COL005.4), a second cousin (COL005.10) and first cousin once removed (COL005.17) with uveal coloboma. Best-corrected visual acuity was 20/15 OD and 20/60- OS (Snellen). Ocular motility was full with small exophorias at distance and near. Pupils were remarkable for a left iris coloboma. Dilated fundus examination showed a linear pigment disturbance inferior to the optic nerve OD (likely a *forme fruste* of coloboma) and a large chorioretinal coloboma OS inferior to the nerve and macula with hyperpigmentation in its periphery. Several years later, a retinal tear developed in the inferior periphery OS that was treated with laser and has remained stable. Systemic examination was remarkable for 11 thoracic vertebrae, mild scoliosis and spina bifida occulta of S1 on spine x-ray. Mitral valve prolapse was noted on ECHO. Kidney ultrasound, brain MRI, bone age, serum cholesterol, vitamin A, karyotype/sub-telomeric FISH and thyroid function were normal. Genome sequencing in two distantly related family members COL005.1 and COL005.10 revealed the same heterozygous 107kb deletion in *NR6A1* (chr9:g.124536516_124643457del, GRCh38). The deletion breakpoints were in intron 2 and 6 removing the coding sequence for amino acids (aa) Ile48-Gly275 and likely causing a frameshift (p.Ile48Asnfs*3, Figure 1H). The status of the heterozygous deletion was determined by breakpoint PCR among family members available, which revealed complete segregation with the missing vertebra with an estimated LOD score of 3.6 (Figure 1A, Figure S1). Four family members were also affected by coloboma in addition to missing vertebra, of which one (COL005.17) also had, by report, only one kidney.

### COL034

The proband of family COL034 (COL034.1) presented at age 11 months with bilateral uveal colobomas, microphthalmia OS (Figure 1E) and a moderate amplitude, low frequency nystagmus with an anomalous head posture. Prenatal history was remarkable for maternal oral contraceptive use at time of pregnancy and an 18-week ultrasound that showed a two-vessel umbilical cord and inability to visualize the left kidney. Delivery was at term and unremarkable with a birth weight of 5lbs, 3oz. Subsequent growth was slow, requiring growth hormone injections. Ocular examination was remarkable for bilateral microcornea (horizontal diameter 9mm OD, 6mm OS), a left iris coloboma, left sensory esotropia, an inferonasal cortical cataract OD, and bilateral chorioretinal and optic nerve colobomas. Best-corrected visual acuity at age 7 was 20/400 OD and <20/800 OS by ETDRS chart. By age 14, the left lens began to dislocate slightly and develop mild/moderate nuclear opacity. Spine x-ray demonstrated 10 thoracic vertebrae. Kidney ultrasound showed a normal right kidney and a missing left kidney (normal retroperitoneal or pelvic). Physical exam was remarkable for curvature to superior prominence to the ears, and a left undescended testicle. Chromosomal microarray was normal. Audiology examination and an echocardiogram were unremarkable. Subsequent endocrine workup showed hypothyroidism requiring replacement (in addition to his growth hormone deficiency). At age 16, he developed intraocular pressures in the low/mid-twenties with a history of slowly progressive vision loss to hand motion, prompting initiation of intraocular pressure lowering drops. A brain MRI at age 17 showed small optic nerves/chiasm, no parenchymal abnormalities, and a normal-appearing pituitary. Genome sequencing revealed a heterozygous c. 274C>T p. (Arg92Trp) variant in *NR6A1*, which was found in the affected mother and essentially unaffected grandfather (Figure 1A, H).

### COL171

The proband of family COL171 (COL171.1) presented at age 36 with bilateral colobomatous microphthalmia affecting the iris, retina/choroid and optic nerve. Visual acuity was 20/640 OD and 20/250 OS. Nystagmus and mild abduction deficits were noted. Slit lamp exam was notable for bilateral microcornea, bilateral posterior subcapsular and nuclear cataracts and missing zonules inferiorly OU (Figure 1F). By report, kidney ultrasound, ECHO and physical exam were normal at birth. Spine x-ray showed normal number and morphology of vertebrae. Audiology examination was notable for mild to moderate sensorineural hearing loss across all frequencies in the right ear and mild sensorineural hearing loss in high frequencies of the left ear. Blood chemistries notable for mild elevation of liver function tests and calcium. Genome sequencing revealed a heterozygous c.1306C>T p.(Arg436Cys) variant in the proband which was absent in his unaffected mother (Figure 1H). Additional family members were not available for segregation analysis, but his father had no history of coloboma by report.

## Supplementary Material

Supplement 1

## Figures and Tables

**Table 1:** Phenotypic details of individuals carrying pathogenic variants in the NEI cohort.

Family	Member*	Age at consent	Sex	Iris coloboma	Retinal/choroidal coloboma	Optic nerve coloboma	Other ocular findings	Vertebral findings	Renal findings	Audiology findings	Other positive medical findings	Negative medical findings
COL005	COL005.1	14	F	OS	OU (OD is forme fruste with a small pigmented area inferior to the disc)	OS (slightly anomalous disc)	Amblyopia requiring patching; strabismus requiring surgery; retinal tear OS requiring laser retinopexy; anterior lens pigment and small lens coloboma OS	11 thoracic vertebrae, spina bifida occulta at S1 and minimal scoliosis	None	Not done	Small jaw and abnormal dentition requiring minor surgeries; mitral valve prolapse; limited left sided proctitis (inflammatory bowel disease), internal hemorrhoids; abnormal contour of the left globe on brain MRI	Normal brain MRI (aside from left globe), serum vitamin A, bone age, cholesterol levels, thyroid function, karyotype
	COL005.2	47	F	None	None	None	Myopia, astigmatism and presbyopia	11 thoracic vertebrae and cervical spondylosis	Not done	Non-congenital low frequency hearing loss by report	History of unexplained relapsing fevers with rash	Normal thyroid function
	COL005.4	11	M	None	OD	OU (OS is forme fruste with an anomalous disk with small inferior crescent)	None	11 thoracic vertebrae, 4 lumbar vertebrae and minimal scoliosis	None	Not done	No further testing done	Normal brain MRI, serum vitamin A, bone age, cholesterol levels, urinalysis
	COL005.8	79	M	None	None	None	BCVA was not corrected to 20/20	11 thoracic vertebrae and scoliosis	None	Not done	No further testing done	No further testing done
	COL005.10	28	F	OU (OD: forme fruste with focaliris transillumination inferiorly)	OU	OU (anomalous discs)	Posterior subcapsular cataract OS; mild heterochromia; history of increased intraocular pressures, treated with latanoprost; bestcorrected visual acuity OS 20/40	Transitional thoracolumbar and lumbosacral vertebral bodies	None	Notched configuration of audiogram in left ear at 2000Hz, with evidence of a slight air-bone gap at that frequency	Abnormal contour of the globes on brain MRI	Normal brain MRI (aside from globes), serum vitamin A, cholesterol levels, serum chemistries, liver function tests, urinalysis, karyotype
	COL005.13	40	F	None	None	None	Myopia	11 thoracic vertebrae and scoliosis of upper thoracic spine	Not done	Not done	History of hypothyroidism	No further testing done
	COL005.17	58	M	Not done	Coloboma (unsp.) per report	Not done	Not done	One missing vertebra per report	Missing kidney per report	Not done	No further testing done	No further testing done
	COL005.18	30	M	None	None	None	Myopia	11 thoracic vertebrae	Not done	Not done	No further testing done	No further testing done
COL034	COL034.1	1	M	OS	OU	OU	Microphthalmia OS; microcornea ou with pigment deposition on endothelium OS; shallow anterior chamber OS with elevated intra ocular pressure; inferonasal cortical cataract OD; moderate amplitude, low frequency nystagmus with an anomalous head posture	10 thoracic vertebrae	Missing left kidney	None	Small size of the optic chiasm and optic nerves, and abnormal contour of the globes on brain MRI; curvature to superior prominence to the ears; history of left undescended testicle; history of growth hormone deficiency and hypothyroidism	Normal chromosomal microarray; normal pituitary gland on pituitary gland MRI; normal echocardiogram
	COL034.2	36	F	None	OS (retinal thinning and pigment inferior to disc)	None	Non-visually significant anterior subcapsular cataracts and anterior pigment OU	11 thoracic vertebrae and spina bifida occulta at L4, L5, and S1	None	Not done	None	Normal serum chemistries
	COL034.6	62	M	None	None	OU (slightly dysplastic and borderline small optic nerves)	Early stage nuclear and cortical cataracts OU; midperipheral temporal patchy deep depigmentation OD, possibly from old infection	Minimal disc space narrowing noted posteriorly at C5–6 with small anterior soft tissue ossification at C5–6, C6–7; minimal degenerative changes in the lumbar spine	Not done	Not done	History of parathyroid surgery; hypercholesterolemia	No further testing done
COL171	COL171.1	36	M	OU	OU	OU	Nystagmus and mild abduction deficits; bilateral microcornea; bilateral posterior subcapsular and nuclear cataracts and missing zonules inferiorly	Mild anterior longitudinal ligament calcification at C5–6 and C6–7 and possible vascular calcification noted within the soft tissues anterior to the L4–5 disc level	None	Mild to moderate sensorineural hearing loss across all frequencies in the right eye and mild sensorineural hearing loss in high frequencies of the left ear	Mild elevation of liver function tests; mild elevation of calcium level on mineral panel	Normal urinalysis

*: Subject numbers are noted in the pedigrees in Figure 1.
