# Supplementary material for: Variants in NR6A1 cause a novel oculo-vertebral-renal (OVR) syndrome
Source: Res Sq. 2024 Nov 15:rs.3.rs-5375105. Preprint. [Version 1] doi: 10.21203/rs.3.rs-5375105/v1 (PMC11601836; doi:10.21203/rs.3.rs-5375105/v1)
Supplement: Supplement 1 [file NIHPPRS5375105V1-suppliment-1.pdf]

Supplementary Methods

Genetic testing

Genomic DNA samples prepared from blood or saliva from NEI patients, and their family members were subjected to short-read Next-generation sequencing (NGS) using Illumina platforms. In total, 101 proband samples were subjected to amplicon sequencing of the *NR6A1* gene (Supplementary Table 2) using a MiSeq sequencer (2 x 300 bp paired-end), 57 samples subjected to exome sequencing (2 x 150 bp paired-end, xGen exome v1 supplemented with additional probes, Blueprint Genetics), 66 samples subjected to GS (2 x 150 bp paired-end, PCR-free library, NIH Intramural Sequencing Center). Reads were aligned to the GRCh38 reference genome, small variants and structural variants were then called, annotated, and prioritized using a custom NGS analysis pipeline (https://github.com/NIH-NEI/NGS_genotype_calling & https://github.com/NIH-NEI/variant_prioritization).

Sanger sequencing was performed to confirm select variants in probands and family members using the BigDye-direct sequencing kit (Thermo Fisher) using primers provided in Supplementary Table 2. The deletion breakpoint in family COL005 was also determined by PCR and Sanger sequencing (Supplementary Table 2). Breakpoint PCR was further used for genotyping of the COL005 family. The logarithm of the odds (LOD) score in family COL005 was estimated using the formula $\log_{10}(1/0.5^{\text{Segregations}})$.

Additional patients and family members underwent Genome Sequencing (GS) as part of the UK100KGP including the clinical variant interpretation pipeline (The National Genomics Research Library v5.1, Genomics England. doi:10.6084/m9.figshare.4530893/7. 2020.). Genome data from affected individuals recruited with a clinical phenotype in keeping with microphthalmia, anophthalmia or coloboma were interrogated for rare (minor allele frequency <0.001, gnomAD v3.1 dataset) biallelic or *de novo* protein altering variants across the genome. Candidate variants underwent manual curation including *in silico* prediction, literature search and pathway analysis to establish biological plausibility as a pathogenic variant in developmental eye disease. Additional analyses of all rare protein altering variants

in *NR6A1* across the entire UK100KGP was performed to identify any individuals outside of the ophthalmology cohort who harbored a candidate pathogenic variant. All variants were manually inspected in the Integrative Genomics Viewer (IGV) after loading sample bam files. Variants appeared to be artifacts were not reported.

Variant classification

The 2015 ACMG/AMP sequence variant interpretation guidelines were followed for variant classification [31,32]. The PM1 (functional domain) criterion was applied to variants in part of the DNA binding domain, a.a. Thr68-Lys119, as the region is highly constrained for missense variations in gnomAD (v2.1.1, missense observed/expected = 0.19, p-value = 6×10^{-6}). The PP3 criterion was applied to missense variants based on a collection of in-house in silico prediction tools (https://github.com/NIH-NEI/variant_prioritization) and the inframe deletion variant based on five in silico prediction tools (CAPICE, FATHMM-indel, MutationTaster, MutPred-Indel, and SIFT.

Molecular modeling

A structural model of NR6A1 was generated using the AlphaFold server, AF-Q15405-F1-model_v4). The Zn-finger domain (ZFD) and nuclear receptor ligand binding domain (NR_LBD) were saved as two PDB files. The binding of DNA to the ZFD of NR6A1 was modeled using a single ZFD domain of the retinoid X receptor alpha-liver X receptor beta (PDB ID: 4NQA) in a complex with DNA. Two variants (R92W and R436C) were generated using the Edit > Swap > Residue function on the respective domain PDB files in YASARA (http://www.yasara.org/). Variant models were optimized and minimized using gradient descent. All two minimized mutants and the two WT, ZFD and NR_LBD models were subjected to 10 ns of Molecular Dynamics (MD) using YASARA's 'run.mcr' macro. Ion concentration was added as a mass fraction with 0.9% NaCl. The simulation temperature was set to 310 K with a water density of 0.997 g/mL. For each domain, the cell size extended to 10 Å beyond each side of the protein in the shape of a cube. Dimensions were 90.2Å x 90.2Å x 90.2 Å and 82.5 Å x 82.6 Å x 82.6 Å for the nuclear receptor Zn-finger and ligand-binding domains, respectively. Each simulation was run in YASARA using an AMBER14 forcefield, with a timestep of 2.5 fs. Simulation snapshots were outputted for every 0.1 ns, resulting in 100 simfiles for each simulation.

Fish maintenance and zebrafish strains

Danio rerio were maintained under standard conditions. Embryos were staged according to Kimmel et al., 1995 [33]. ABTL stocks were used for all the experiments, which were carried out in accordance with National Eye Institute, Animal Care and Use Committee Protocol Number NEI-648.

Zebrafish *in situ* hybridization

Embryo were fixed in 4% paraformaldehyde (PFA) overnight at 4°C and dehydrated in methanol for 1h at −30°C. The embryos were rehydrated, treated with proteinase-K and re-fixed with 4% PFA. Pre-hybridization and hybridization were carried out at 65°C. RNA probes were synthesized using a DIG labeling kit (Millipore-Sigma, 112770739) following manufacturer's protocol. *nr6a1a* RNA probe was synthesized from a CDS clone in TOPO TA vector (ThermoFischer Scientific), while *nr6a1b* was synthesized using PCR product as a template. Primers are noted in Supplementary Table 3. Samples were hybridized overnight with RNA probes at 65°C, washed, incubated with Anti-DIG antibody (Millipore-Sigma,

1109327490); color was developed using BCIP/NBT substrate (Millipore-Sigma, 11681451001) in alkaline phosphatase buffer. Embryos were imaged with Leica DM6 dissecting microscope.

Morpholino gene knockdown and rescue experiments in zebrafish

All morpholinos (MO) were obtained from Gene Tools LLC. MOs used to target zebrafish *nr6a1a* and *nr6a1b* are given in Supplementary Table 4. Human *NR6A1*-wild type, variants *NR6A1*-R92W and *NR6A1*-R436C DNA fragments were synthesized and cloned in pCS2+ (Azenta Life Sciences). Plasmids were linearized with *Not I* restriction enzyme and capped mRNA was synthesized using mMessage mMachine T7 Transcription kit (ThermoFischer Scientific). MOs and mRNA were co-injected into zebrafish embryos at single cell stage. *nr6a1a* and *nr6a1b* translation blocking (TB) MOs were used at 2ng and 1.25ng respectively. Nr6a1a and nr6a1b, SB-MOs were injected at 2ng and 1ng respectively. Human *NR6A1*-wild type was used at 100pg and 150-200pg for RNA rescue and over expression studies respectively. *NR6A1*-R92W and *NR6A1*-R436C RNAs were used at 100pg for rescue experiments. For over-expression experiments, doses of 100pg-200pg *hNR6A1* mRNA were injected at the single cell stage. Embryo phenotypes were scored and imaged at 72 hours post-fertilization (hpf) using Leica DM6 dissecting microscope.

Cell culture and transfection studies

HEK293T cells maintained in DMEM with 10% FBS and 1% penicillin-streptomycin were seeded onto 4-well chamber slides, maintained for 24 hr and transiently transfected with GFP tagged WT and/or mutant *NR6A1* constructs (Azenta Life Science, Burlington, MA, USA) using X-treme Gene HP (Roche, Indianapolis, IN, USA) following manufacturers' instructions. After 24-48 hrs of transfection, transfected cells were fixed for 15 mins in 4% paraformaldehyde (PFA) in PBS. After washing with 1× PBS cells were incubated for 1 hr at room temperature with Hoechst33342 (1:250 dilution in PBST). Subsequently, the slides were washed and mounted with Fluoromount-G® (SouthernBiotech, Birmingham, AL, USA). Zeiss confocal microscopes 880 coupled with an Airyscan® detector was used for confocal imaging. The images were analyzed using ZEN Software (Carl Zeiss Microscopy LLC, Thornwood, NY). The cell culture experiments were repeated at least three times for each for variant localization studies.

Flow cytometry

Transfection efficiency was determined by measuring the expression of GFP after 48 hrs post transfection. HEK293 cells were detached from the plates using Trypsin for 5 mins followed by neutralization with serum containing media. The cells were then fixed for 15 mins in 4% paraformaldehyde (PFA) in PBS and then collected in 1xPBS containing 2% FBS (FACS buffer) and washed 2 times by centrifugation. The cell suspension was filtered through a 50 µm cell strainer. Data was acquired with a CytoFlex NUV instrument (Beckman Coulter, Brea CA) using the blue light excitation and 525 nm emission to detect GFP and violet light excitation and 450 nm emission to detect DAPI detection. Data analysis was done using CytExpert software Version 2.5 (Beckman Coulter, Brea CA). Interesting cells were identified as DAPI negative, in the whole cell cluster in a FSC vs. SSC plot and being in a single cell state in the FSC-A vs. FSC-Width. Transfection efficiency was quantified as the Stain Index of GFP fluorescence intensity, which was calculated using the median fluorescent intensity and robust Standard Deviation as described.14 The cell culture experiments were repeated at least three times for each for variant localization studies.

Mouse Embryo *in situ* Hybridization

Nr6a1 mRNA expression in mouse was assayed by RNA *in situ* hybridization with *Nr6a1*(Cat: 1314941-C1) probe using the RNAScope Assay, Multiplex fluorescent Reagent Kit V2 (Advanced Cell Diagnostics (ACD), Newark, CA, USA) on E10.5 and E11.5 cryosection as previously described [34].

Gene expression analysis of *NR6A1*

The h5ad (d27a79a1-8a5f-404d-8063-52e19122ef49.h5ad for adult and 88444d73-7f55-4a62-bcfe-e929878c6c78.h5ad for fetal) from the HRCA project were downloaded from cellxgene.cziscience.com and the raw counts were summed at the sample and cell type level to create a pseudobulk matrix with the python package ADPBulk (https://github.com/noamteyssier/adpbulk). The eyeIntegration (which includes GTEx) gene counts and metadata were downloaded from eyeIntegration.nei.nih.gov (https://hpc.nih.gov/~mcgaugheyd/eyeIntegration/2023/gene_counts.csv.gz and https://hpc.nih.gov/~mcgaugheyd/eyeIntegration/2023/eyeIntegration23_meta_2023_09_01.built.csv.gz).

The pseudobulk and bulk RNA-seq counts were normalized with by CPM and transformed in R/4.3 to have a mean of zero and a standard deviation of one. The first four principal components were removed with the WGCNA tool removePrincipalComponents. The correlation matrix was created with the base R cor function. The correlation scores were expression transformed with the spqn package's normalize correlation function. Plots of the expression of NR6A1 were created in R/4.3 with the ggplot2, cowplot, and ggbeeswarm packages.

Supplementary Tables

[See Excel Spreadsheet]

Supplementary **Table 1:** Variant and phenotypic information of rare NR6A1 variant carriers in the UK100KGP cohort

Supplementary **Table 2**: PCR primers used for human DNA sequencing

Supplementary **Table 3:** Primers used in zebrafish in situ experiments

Supplementary **Table 4:** Morpholinos used in zebrafish gene knock-down experiments.

Supplementary **Table 5:** Detailed breakdown of zebrafish morpholino experiments

Supplementary Figures and Legends

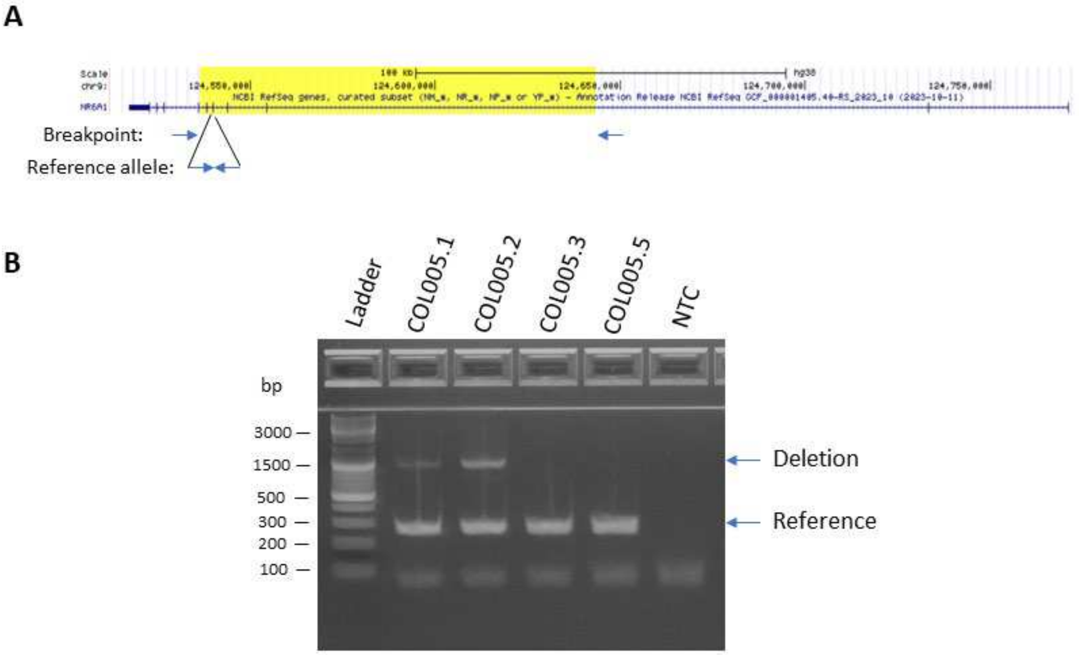

<u>**Supplementary Fig. 1**</u>**: NR6A1 genotyping for the deletion in family COL005** (A), Diagram of the 107-kb deletion (yellow-highlighted) and primer binding sites. (B), Example image of the agarose gel electrophoresis of duplex PCR.

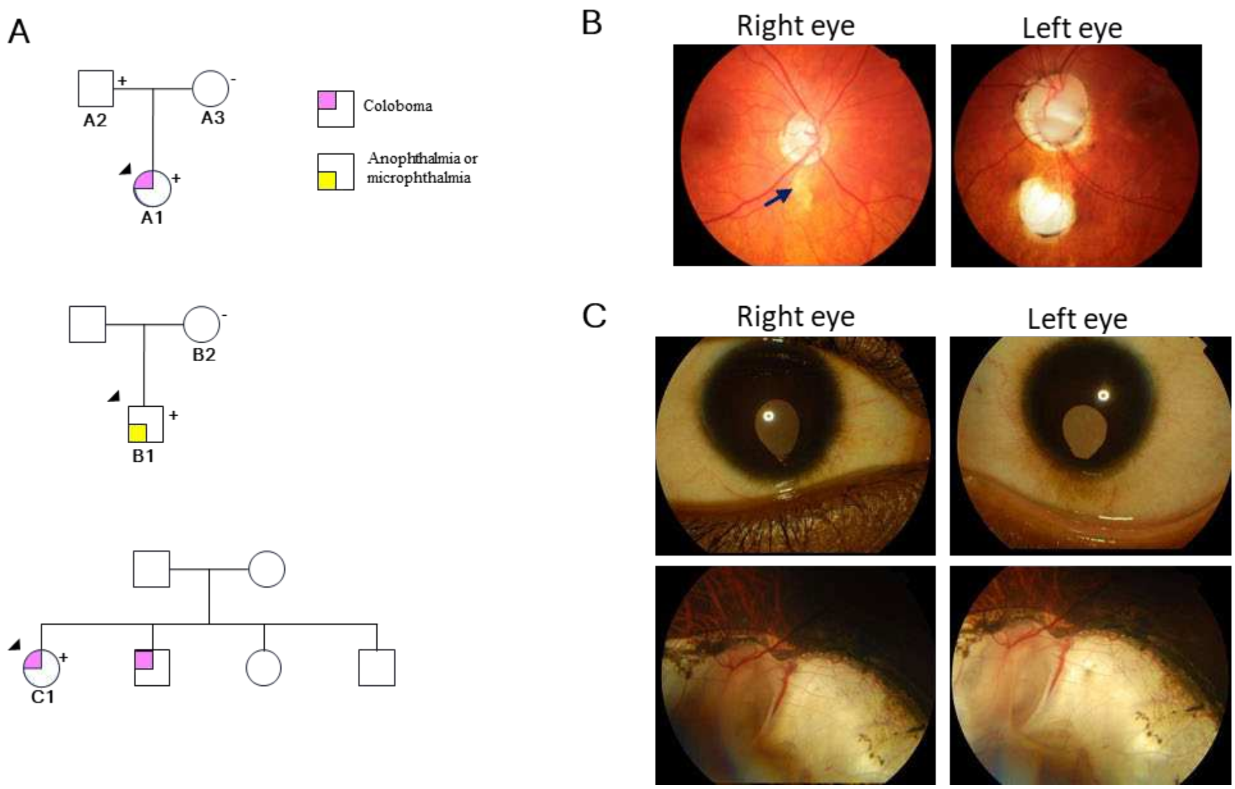

<u>Supplementary Fig. 2:</u> Phenotypes associated with *NR6A1* variants in UK100KGP. A. Pedigrees of three families (A; B; C) from the UK100KGP cohort demonstrating coloboma with or without microphthalmia. Inheritance is autosomal dominant with incomplete penetrance and variable expressivity. **B.** Chorioretinal coloboma found in individual A1; *forme fruste* on the right eye (arrow). **C**. Iris coloboma and chorioretinal coloboma found in individual C1. +, individual with variant; -, individual without variant. See Supplementary Table 1 for variant information.

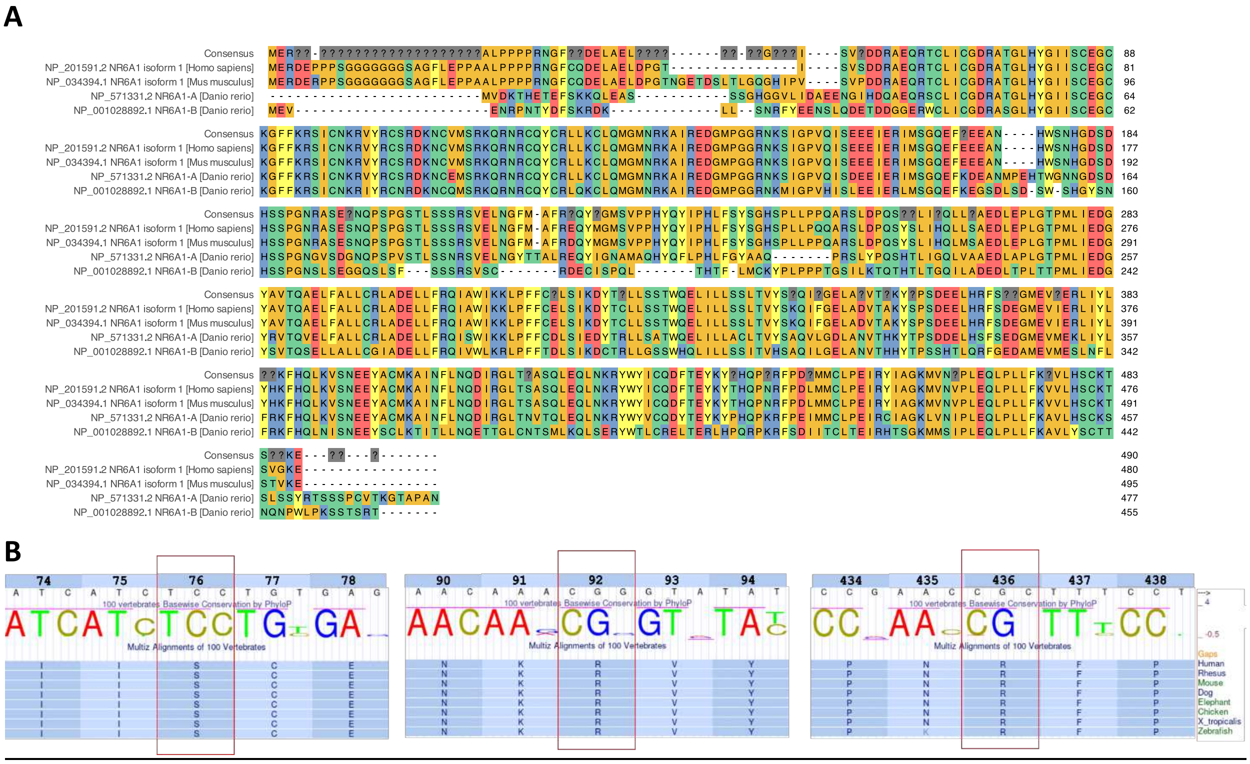

<u>Supplementary Fig. 3</u>: NR6A1 conservation A. Multiple species alignment (MSA) of the canonical transcript of human, mouse, and zebrafish *NR6A1*/*Nr6a1*/*nr6a1* (a and b paralogs) showing a high degree of conservation. **B.** Conservation of Ser76, Arg92 and Arg436 residues during vertebrate evolution. The images for 100 vertebrates Basewise Conservation by PhyloP and Multiz Alignments of 100 Vertebrates were adapted from the UCSC genome browser. Human NR6A1 cDNA sequence and amino acid numbers are listed on the top.

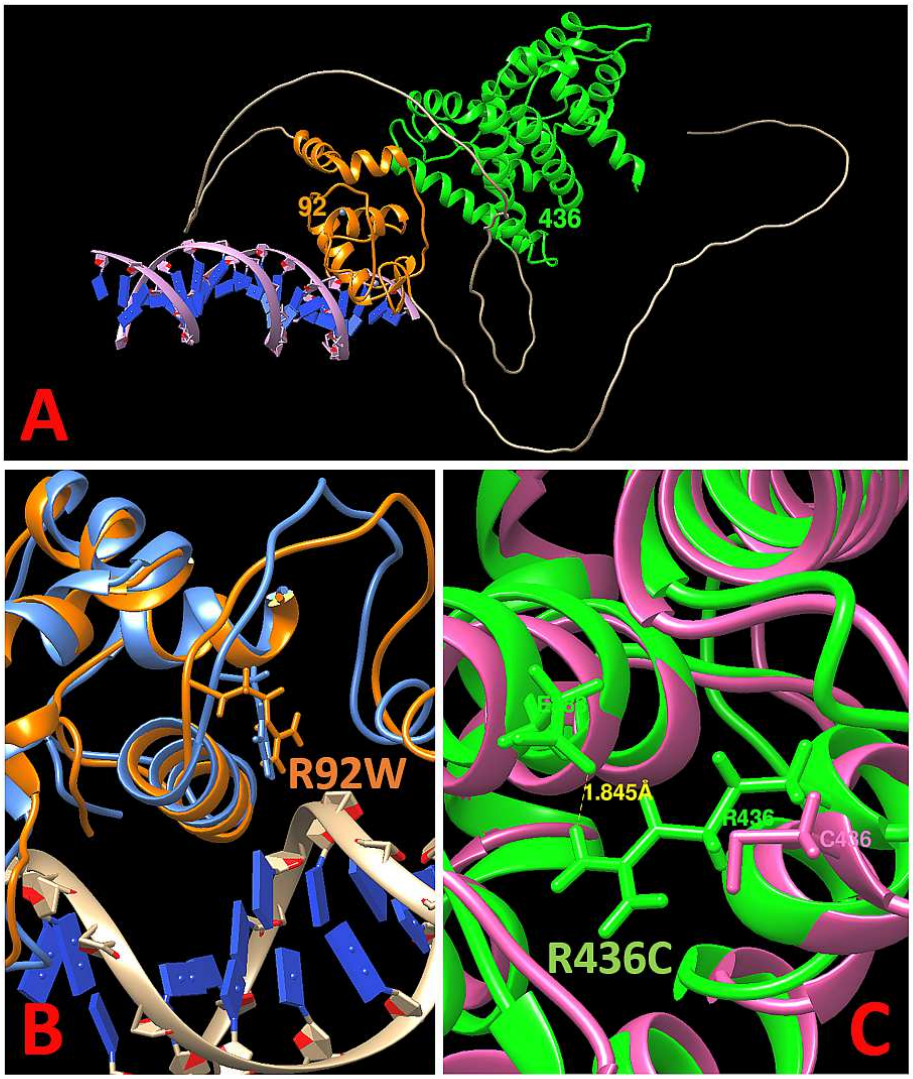

<u>Supplementary Fig. 4:</u> **A.** ***In silico*** **molecular modeling of NR6A1 with DNA** (lilac helix with blue base pairs). The predicted DNA-binding domain and ligand-binding domain are shown in orange and green, respectively. The positions of R92W and R436 are noted. **B**. The R92W variant changes a positively charged Arg to a hydrophobic Trp and is expected to disrupt interaction with the negatively charged DNA helix. **C.** The R436C variant is predicted to disrupt a hydrogen bond between R436 and E388 and substitute a Cys residue that could form abnormal disulfide bridges within the protein.

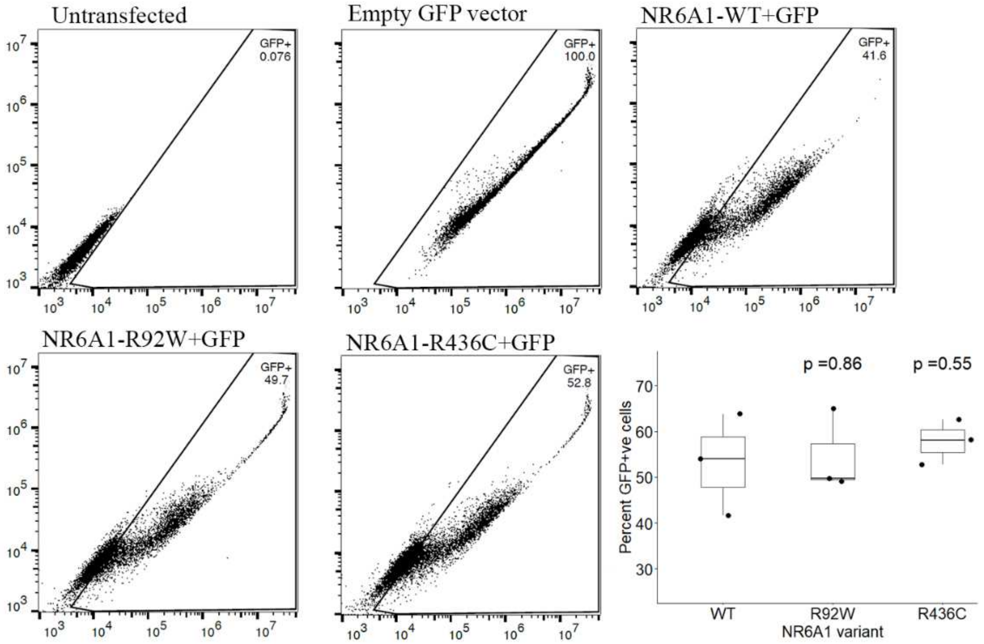

Supplementary Fig. 5: Flow cytometry of wild-type (WT) and mutant (R92W, R436C) forms of NR6A1 demonstrating comparable transfection efficiencies.

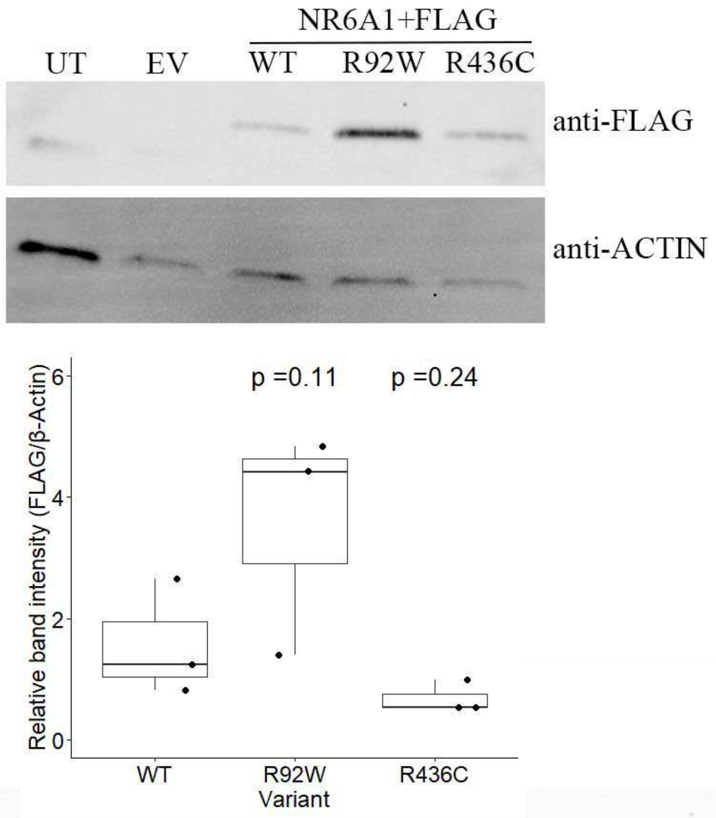

Supplementary Fig. 6: Western blot of HEK293 cells transfected with wild-type (WT) or mutant (R92W, R436W) forms of *NR6A1*, demonstrating comparable transfection efficiencies.

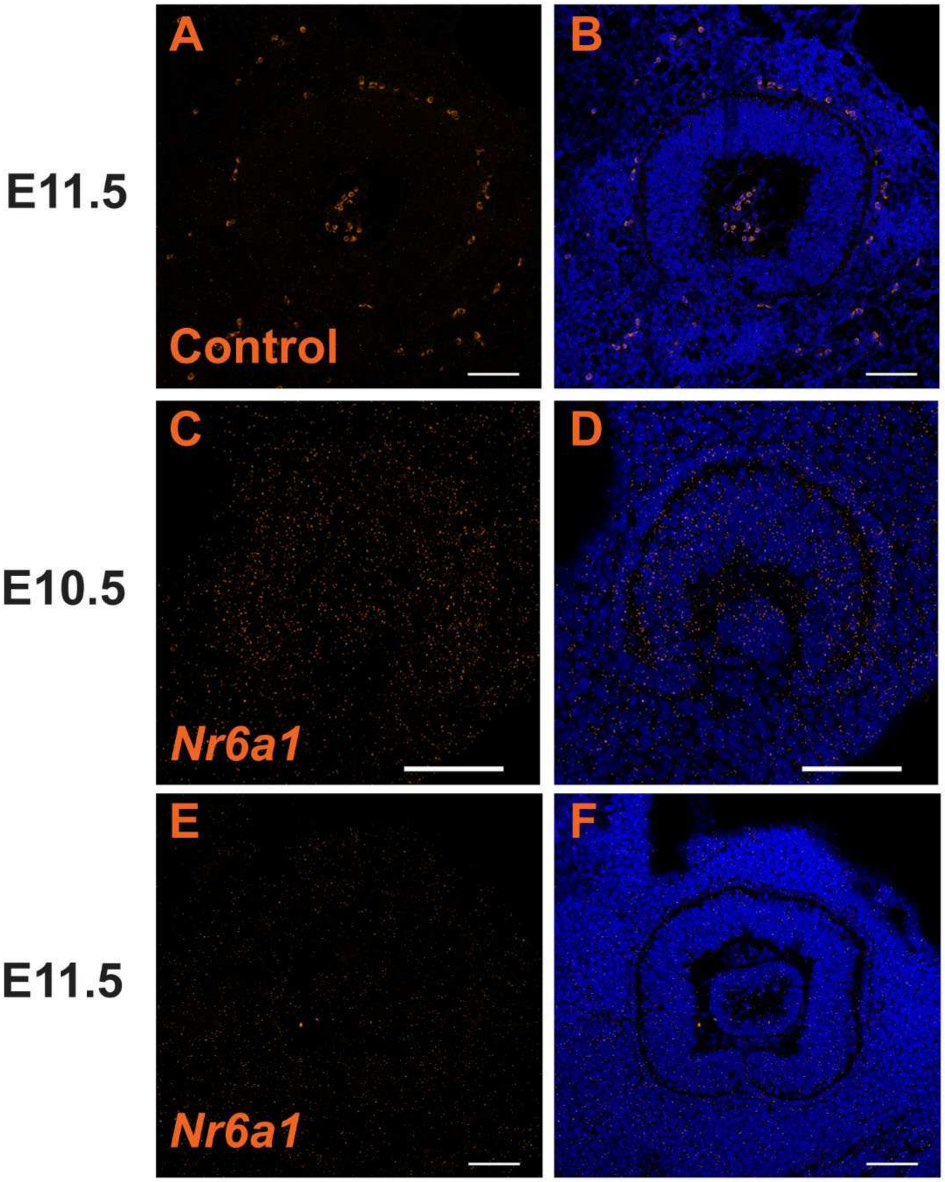

<u>Supplementary Fig. 7:</u> Expression of *Nr6a1* in sagittal sections of mouse embryonic eye before (E10.5) and during (E11.5) optic fissure closure. Low level expression throughout the tissue at E10.5 (C,D) becomes significantly downregulated by E11.5. (E,F). Expression shown with and without DAPI counterstain along with control samples (A,B). Scale bar = 100 µm.

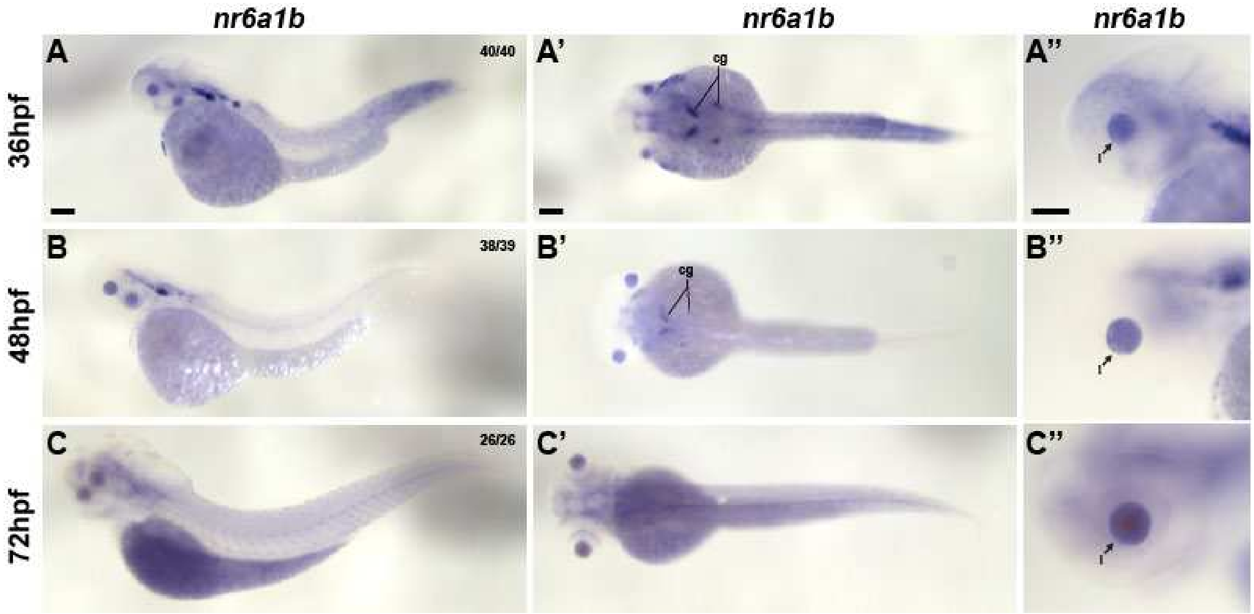

Supplementary Fig. 8: *nr6a1b* mRNA expression during later developmental stages (36 hpf (A-A''), 48 hpf (B-B''), and 72 hpf (C-C'')) of zebrafish development. Note prominent expression in the developing lens (arrow) at all time points and decreased expression in the somites and neural tube, compared to earlier time points. cg-cranial ganglia, l-lens. Scale bar = 100 µM.

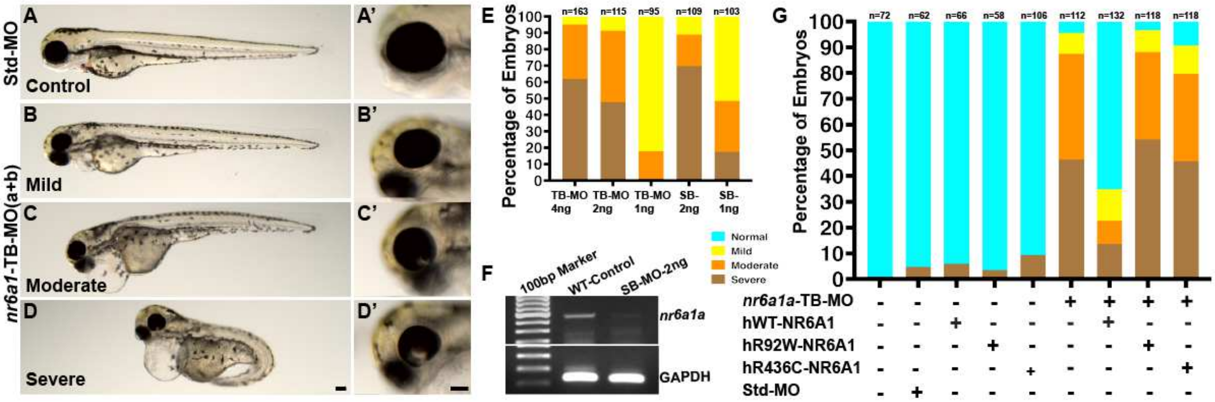

<u>Supplementary Fig. 9</u>: Morpholino (MO) mediated knockdown of the *nr6a1a* paralog with either translation blocking (TB-MO) or splice-blocking (SB-MO) results in a spectrum of phenotypes including colobomatous microphthalmia, shortened/bent body axis and heart edema (A-D, with higher magnification of the eye in the corresponding A'-D' panel). 2ng of either TB-MO or SB-MO was sufficient to cause 80-90% of embryos to have a moderate or severe phenotype (E). Efficient blocking of the splicing by the SB-MO was confirmed with reverse-transcriptase PCR (F). Although 100pg human wild-type (hWT) mRNA resulted in dramatic phenotypic rescue of the TB-MO (>60% with a normal phenotype), rescue with either hR92W- or hR436W-mRNA was considerably less effective (F). Scale bar = 100µM.

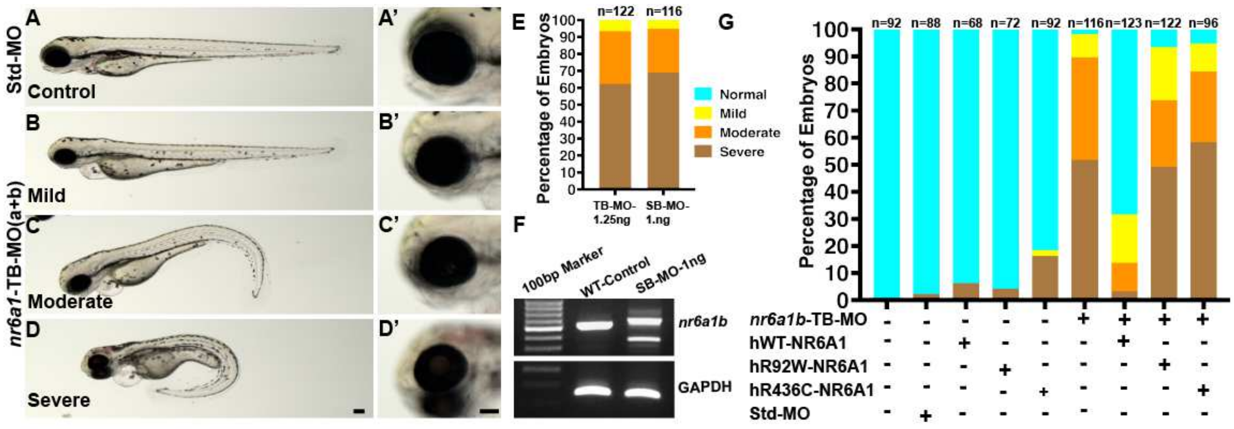

Supplementary Fig. 10: Morpholino mediated (MO) knockdown of the *nr6a1b* paralog with either translation blocking (TB-MO) or splice-blocking (SB-MO) results in a spectrum of phenotypes including microphthalmia, shortened/bent body axis and heart edema (A-D, with higher magnification of the eye in the corresponding A'-D' panel). 1.25ng and 1ng of TB-MO and SB-MO respectively, was sufficient to cause 80-90% of embryos to have a moderate or severe phenotype (E). Efficient blocking of the splicing by the SB-MO was confirmed with reverse-transcriptase PCR (F). Although 100pg human wild-type (hWT) mRNA resulted in dramatic phenotypic rescue of the TB-MO (>60% with a normal phenotype), rescue with either hR92W- or hR436W-mRNA was considerably less effective (F). Scale bar = 100µM.

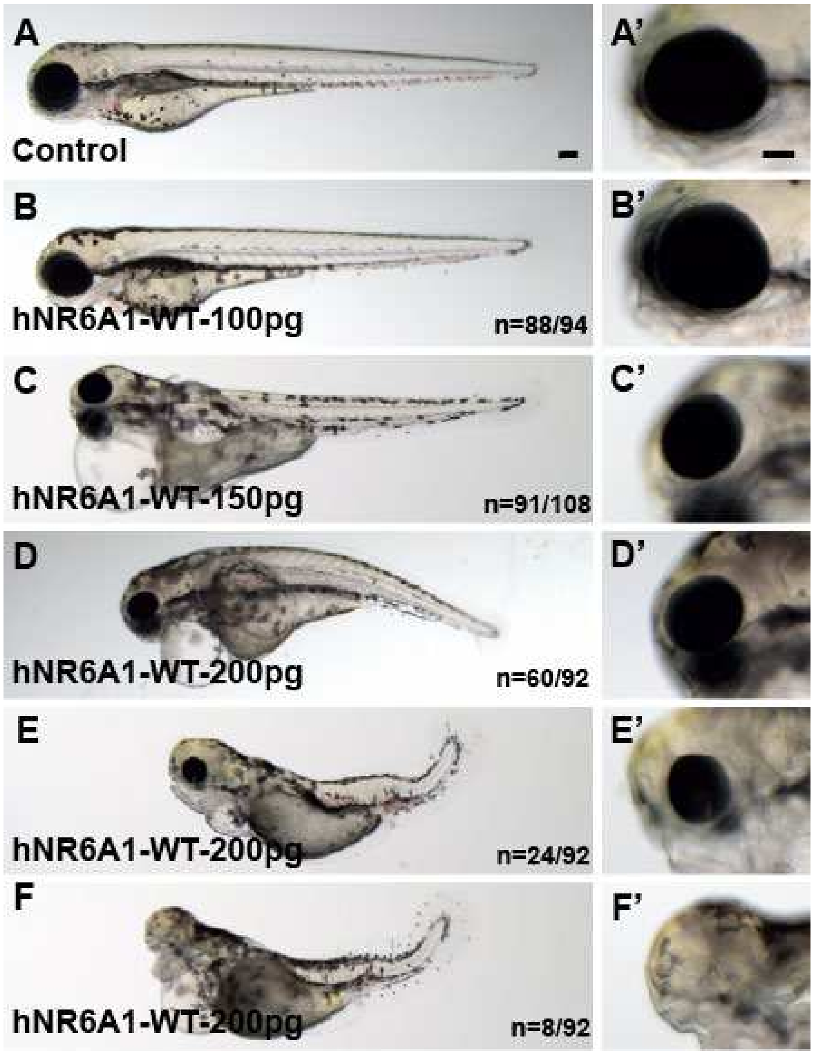

<u>Supplementary Fig. 11</u>: Overexpression of human NR6A1 (*hNR6A1*) in zebrafish: Control and 100pg injected embryos have a normal body with closed OF (A-B'). Embryos injected with 150pg of *hNR6A1* have a shortened but straight body axis, microphthalmia and heart edema (C, C'). A variable phenotype is observed with 200pg of *hNR6A1* RNA, including a bent body axis, heart edema and colobomatous microphthalmia (D, D') The remaining embryos have a curved body axis with somites losing their chevron shape, microphthalmia with coloboma, and in some cases, lack of eyes and heart edema (E-F'). WT = wild type. Scale bar = 100µM.

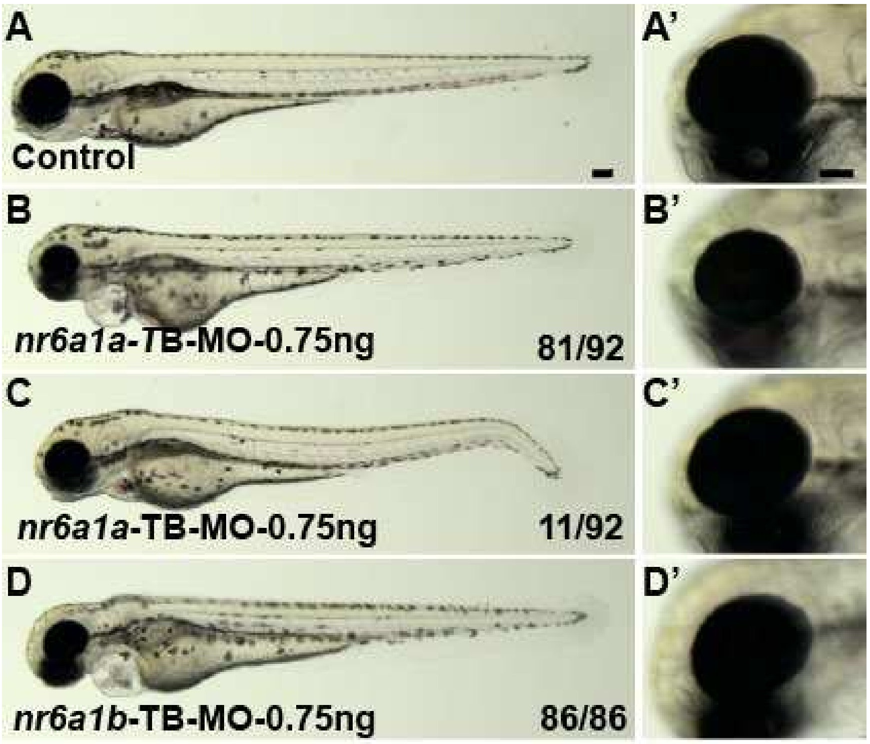

<u>Supplementary Fig. 12</u>: Effect of individual TB-MO for *nr6a1a* and *nr6a1b* at 0.75ng. Control embryos have a straight body and a closed OF (A, A'). Both *nr6a1a* and *nr6a1b* TB morphants at 0.75ng dosage, had straight bodies and microphthalmia, except a few embryos from the *nr6a1a* group which had a slightly curved tail (B-D'). Scale bar = 100µM.

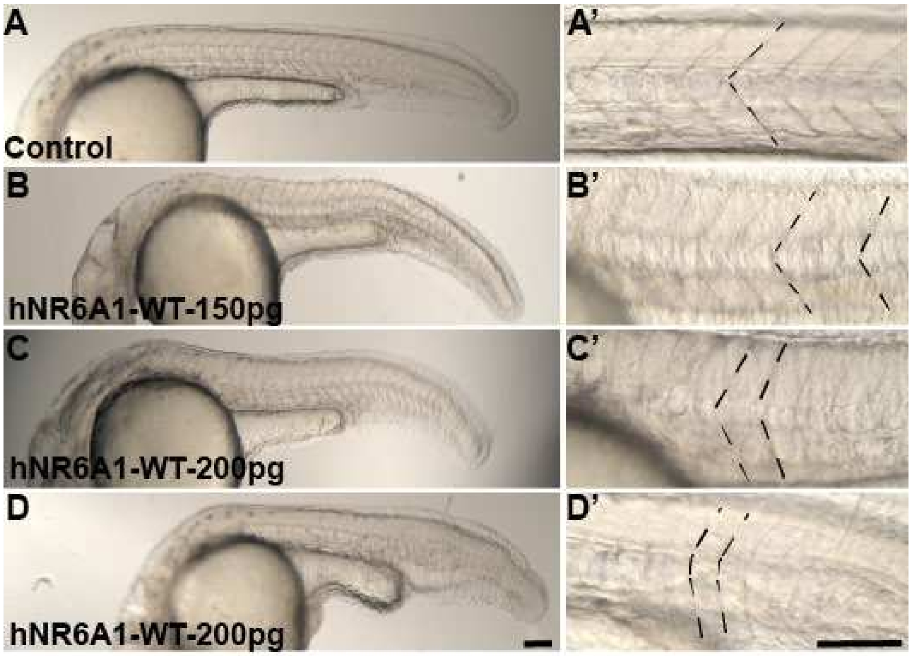

<u>Supplementary Fig. 13</u>: Phenotype of somites in zebrafish embryos over expressed with human NR6A1 (*hNR6A1)*: Control embryos have chevron shaped somites (A, A'), embryos injected with 150pg and 200pf of *hNR6A1* lose the shape of their somites at varying degrees (B-D"). Scale bar = 100µM.

Supplementary Files

This is a list of supplementary files associated with this preprint. Click to download.

- SupplementalTables.xlsx
